# Zoonosis and zooanthroponosis of emerging respiratory viruses

**DOI:** 10.3389/fcimb.2023.1232772

**Published:** 2024-01-05

**Authors:** Ahmed Magdy Khalil, Luis Martinez-Sobrido, Ahmed Mostafa

**Affiliations:** ^1^ Disease Intervention & Prevention and Host Pathogen Interactions Programs, Texas Biomedical Research Institute, San Antonio, TX, United States; ^2^ Department of Zoonotic Diseases, Faculty of Veterinary Medicine, Zagazig University, Zagazig, Egypt; ^3^ Center of Scientific Excellence for Influenza Viruses, Water Pollution Research Department, Environment and Climate Change Research Institute, National Research Centre, Giza, Egypt

**Keywords:** zoonosis, zooanthroponosis, pandemic, swine influenza, COVID-19, respiratory viruses

## Abstract

Lung infections in Influenza-Like Illness (ILI) are triggered by a variety of respiratory viruses. All human pandemics have been caused by the members of two major virus families, namely *Orthomyxoviridae* (influenza A viruses (IAVs); subtypes H1N1, H2N2, and H3N2) and *Coronaviridae* (severe acute respiratory syndrome coronavirus 2, SARS−CoV−2). These viruses acquired some adaptive changes in a known intermediate host including domestic birds (IAVs) or unknown intermediate host (SARS-CoV-2) following transmission from their natural reservoirs (e.g. migratory birds or bats, respectively). Verily, these acquired adaptive substitutions facilitated crossing species barriers by these viruses to infect humans in a phenomenon that is known as zoonosis. Besides, these adaptive substitutions aided the variant strain to transmit horizontally to other contact non-human animal species including pets and wild animals (zooanthroponosis). Herein we discuss the main zoonotic and reverse-zoonosis events that occurred during the last two pandemics of influenza A/H1N1 and SARS-CoV-2. We also highlight the impact of interspecies transmission of these pandemic viruses on virus evolution and possible prophylactic and therapeutic interventions. Based on information available and presented in this review article, it is important to close monitoring viral zoonosis and viral reverse zoonosis of pandemic strains within a One-Health and One-World approach to mitigate their unforeseen risks, such as virus evolution and resistance to limited prophylactic and therapeutic interventions.

## 1 Introduction

Zoonotic viral pathogens are those pathogens that can escape species barriers to transmit or jump from their non-human natural reservoirs, including avian or mammalian species, to humans in a process that is known as zoonosis. Most human infectious diseases (60-75%) are derived from pathogens that originally circulated in non-human animal species (Ellwanger and Chies, 2021). The ability of the virus to escape species barriers and jump to infect humans is always associated with hazardous consequences on individual and public health due to the lack of pre-existing immunity to the invading zoonotic virus, representing unforeseeable health concern (Seal et al., 2021; Tomori and Oluwayelu, 2023). Zoonotic viruses may occasionally infect humans and can cause diseases in people ranging from mild to severe symptoms and even death (Mostafa et al., 2018; Rahman et al., 2020). During this century, the world has been confronted with the emergence of two respiratory pandemics that were originally transmitted from animals to human, specifically influenza A/H1N1 in 2009 and coronavirus disease 2019 (COVID-19), caused by the 2009 influenza H1N1 virus (A/H1N1pdm09) and severe acute respiratory syndrome coronavirus 2 (SARS-CoV-2), respectively. In this narrative review article, we review these human respiratory virus pandemics, their frequent host-jumping events between human and non-human animal species, and the molecular determinants that ease viral transmission or improve viral fitness in domestic pets and wildlife animals, including the potential of establishing new vessels for virus evolution and spreading. In addition, the ability of influenza A/H1N1pdm09 virus and SARS-CoV-2 to infect other hosts with diverse biological factors is usually associated with the emergence of immune escape or drug-resistant variant(s). Hereafter, we also discuss the impact of the interspecies circulation of the two pandemic viruses on the currently available medical interventions.

## 2 Origin and zoonotic potential of influenza and coronaviruses

Influenza viruses are single-stranded, negative-sense segmented enveloped RNA viruses that belong to the family *Orthomyxoviridae* in the order Mononegavirales (Webster et al., 1992), and are divided in four types: A, B, C, and D. While influenza A (IAV) and B (IBV) viruses infect humans and induce seasonal epidemics with occasional pandemics, influenza C viruses (ICV) can infrequently infect humans with mild cold-like symptoms especially in young children (Calvo et al., 2006), and influenza D viruses (IDV) mainly infect cattle, pigs (Liu et al., 2020), and occasionally poultry species (Bailey et al., 2020), with few recently reported human cases in dairy farm workers (Leibler et al., 2023).

Genetically, the IAV particle is composed of a host-derived lipid bilayer envelope with protruding surface glycoproteins, namely hemagglutinin (HA) and neuraminidase (NA), that are encoded by viral segments 4 and 6, respectively. The viral segment 7 encodes two viral proteins, the matrix protein 1 (M1, lining the inner surface of the viral particle) and the matrix protein 2 (M2, transmembrane channels). The core of the viral virion is made of the eight viral ribonucleoprotein complexes (vRNPs) consisting of each viral RNA segment encapsulated by the viral nucleoprotein (NP, encoded by viral segment 5), and containing the three polymerase subunits (polymerase basic 2 (PB2, encoded by viral segment 1), polymerase basic 1 (PB1, encoded by viral segment 2), and polymerase acidic (PA, encoded by viral segment 3)). The 8^th^ viral segment encodes two viral proteins, namely the non-structural protein 1 (NS1) and the nuclear export protein (NEP), or non-structural protein (NS2) (Mostafa et al., 2018).

**Figure 1:**
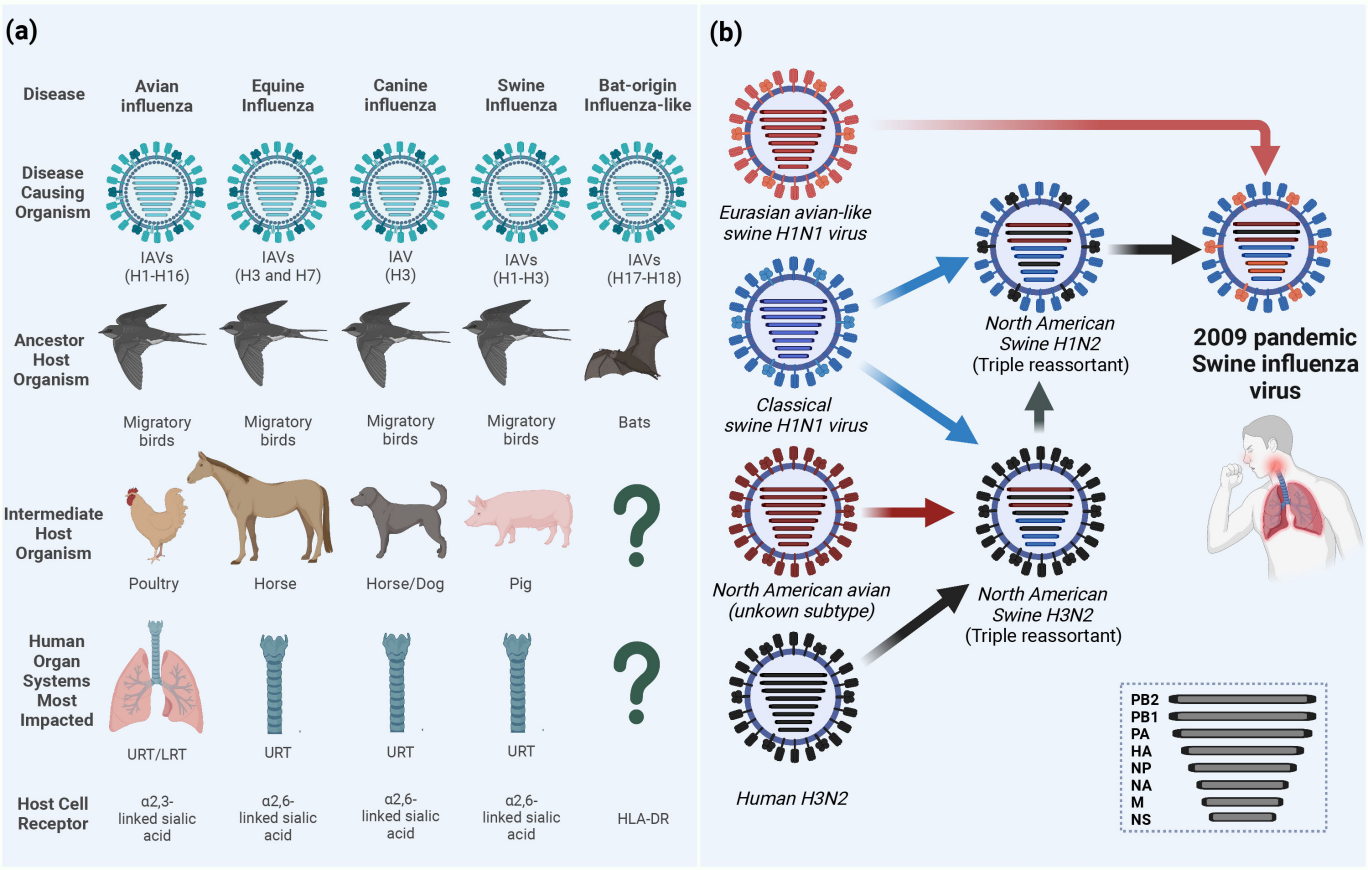
Ecology of influenza A viruses (IAVs) and origin of the 2009 pandemic influenza virus (A/H1N1pdm09). **(A)** IAVs are categorized according to their intermediate or ancestor animal host species into avian influenza viruses (AIVs), equine influenza viruses (EIVs), canine influenza viruses (CIVs), swine influenza viruses (SIVs) or bat-origin influenza-like viruses (BIVs). Unlike all AIVs, EIVs, CIVs and SIVs that can bind sialic acid (SA) receptors on the surface of the host cell and lead to upper respiratory tract (URT) and severe lower respiratory tract (LRT) infections in humans, BIVs do not have the ability to bind SA receptors and rather utilize the major histocompatibility complex class II (MHC-II) human leukocyte antigen DR isotype (HLA-DR) as an entry determinant to the host cells (Karakus et al., 2019). **(B)** Schematic illustration of the reassortments events that lead to the origin of influenza A/H1N1pdm09 virus with unique genetic constellation. Question marks indicate the unknown intermediate host organism or the human organ system most impacted. This figure was created with BioRender.com.

IAVs have a wide host range including humans, equine, canine, swine, and domestic and wild birds (**Figure 1A**). Wild aquatic birds are the major natural reservoirs of IAVs (Mostafa et al., 2018; Rahman et al., 2020). Based on the antigenicity of the two outer surface glycoproteins (HA and NA), avian IAVs (AIVs) are classified into 16 HA and 9 NA subtypes, in addition to two other subtypes H17N10 and H18N11 identified in bats (**Figure 1A**) (Webster et al., 1982; Yang et al., 2021). Due to the complexity in its ecology and genetic nature, IAVs continuously undergo viral evolution that includes both gradual minor (antigenic drift) and sudden major (antigenic shift) changes in the viral genome (Kim et al., 2018).

To date, several zoonotic IAVs were able to cross the species barriers and result in human infections. For instance, IAVs circulating in birds, the so-called AIVs, of H5N1 (Chan, 2002), H5N6 (WHO, 2022), H5N8 (Pyankova et al., 2021), H6N1, H7N2 (Philippon et al., 2020), H7N3 (Freidl et al., 2014), H7N4 (WHO, 2022), H7N7 (Freidl et al., 2014), H7N9 (Petersen et al., 2018; WHO, 2022), H9N2 (Peacock et al., 2019), H10N3 (WHO, 2023a), H10N7, and H10N8 (Philippon et al., 2020) subtypes were reported to infect humans. Unlike AIVs, neither equine influenza virus (EIV), including H3N8 or H7N7 subtypes (Yondon et al., 2014; Chambers, 2022), nor canine influenza virus (CIV) H3N8 or H3N2 subtypes (Martinez-Sobrido et al., 2020) were isolated from humans. However, several serological evidence for equine-to-human transmissions have been reported in humans in different countries (Khurelbaatar et al., 2014; Xie et al., 2016). On the same hand, avian-origin reassortant influenza A/H3N8, expressing the internal proteins-encoding segments from Eurasian lineage A/H9N2 poultry viruses, has been recently detected in an infected boy from China (Bao et al., 2022; Yang et al., 2022). More recently, three human infections with avian influenza A/H3N8 were reported to the World Health Organization (WHO) from China (WHO, 2023b).

Another major host for zoonotic potential is swine which is considered as a mixing vessel for the generation of new genotypes/phenotypes of IAVs. Binding to the correct host cell receptor is the key of establishing virus infection (Schmier et al., 2015). While AIVs and human IAVs preferentially bind to sialic acid (SA) α-linked at C2 to a galactose of cellular glycoprotein at C3 (α2-3 SA) or C6 (α2-6 SA) receptors, respectively, swine has both avian and human receptors whereby it can be infected with both IAVs and generate new subtypes through genetic reassortment between IAVs from different origins (Rogers and D'Souza, 1989). The influenza virus pandemic in 2009 is a paradigm of the genetic reassortment where the genetic segments of IAVs from different sources (human, avian, and swine) mixed in swine to generate the swine-origin IAV (referred to as influenza A/H1N1pdm09 virus) to which humans had no pre-existing immunity (**Figure 1B**) (Smith et al., 2009). To this point, the molecular features and host adaptive substitutions of the influenza A/H1N1pdm09 virus are variable due to the complexity in the genotyping of the emerged virus that resulted from the intermixing of different genes from the North American triple reassortant swine influenza viruses (SIVs) and European avian-like SIVs. For instance, in influenza A/H1N1pdm09 virus, the PB2 and PA genes are from avian origin, the PB1 from human-origin, and the HA, NP, and NS from classical SIVs that altogether came from North American triple reassortant swine influenza; whereas the NA and M genes were acquired from the European avian-like SIV (**Figure 1B**) (Smith et al., 2009). To cross species barrier, several adaptive substitutions were acquired in influenza A/H1N1pdm09 virus to be transmitted from swine and induce infection in humans; then other adaptive substitutions were acquired while circulating in humans. Herein, we provide insights on the different adaptive substitutions in different genes of influenza A/H1N1pdm09 virus that render human infection and continuous circulation. During the first year of virus circulation, influenza A/H1N1pdm09 virus was responsible of 151,700 – 575,400 deaths worldwide (Juvet et al., 2021). Currently, influenza A/H1N1pdm09 viruses circulate and induce epidemics in humans as one of the seasonal influenza virus strains.

**Figure 2:**
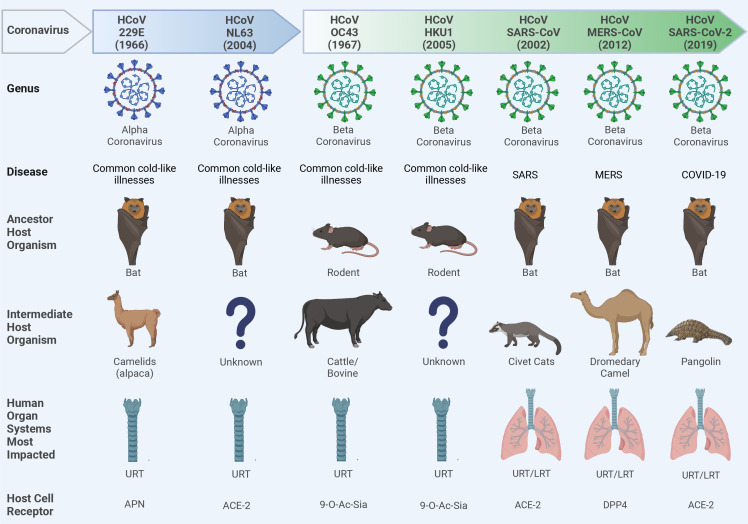
Ecology of human coronaviruses (CoVs). Seven CoVs that belong to alpha-CoVs (229E and NL63) and beta-CoVs (OC43, HKU1, SARS-CoV, MERS-CoV, and SARS-CoV-2) genera could escape species barriers to infect humans following non-hygienic contact with the intermediate host. These CoVs can establish upper respiratory tract (URT) mild infection via binding to different host cell receptors including the amino peptidase N (APN) receptor for 229E; the angiotensin converting enzyme 2 (ACE2) for NL63, SARS-CoV, and SARS-CoV-2; the 9-O-acetylated sialic acid (9-O-Ac-Sia) receptor for OC43 and HKU1; and the dipeptidyl peptidase-4 (DPP4) for MERS-CoV. In severe infections like severe acute respiratory syndrome CoV (SARS-CoV), Middle East respiratory syndrome CoV (MERS-CoV), or Coronavirus Disease 2019 (COVID-19), a lower respiratory tract (LRT) infection can also be developed leading to severe pneumonia and acute respiratory distress syndrome (ARDS). Question marks indicate the unknown intermediate host organisms. This figure was created with BioRender.com.

Coronaviruses (CoVs) are single-stranded, positive-sense enveloped RNA viruses that belong to the family *Coronaviridae* in the order Nidovirales (Weiss and Navas-Martin, 2005). CoVs are classified based on differences in protein sequences into four genera: alphacoronavirus (alpha-CoV), betacoronavirus (beta-CoV), gammacoronavirus (gamma-CoV), and deltacoronavirus (delta-CoV). Beta-CoV are further subdivided into four subgroups (A, B, C, and D) (Su et al., 2016). Based on phylogenetic analysis, rodents are considered the reservoir for many alpha-CoV and beta-CoV, while birds are the main reservoir for gamma-CoV and delta-CoV (Su et al., 2016). To date, seven CoVs jumped the species barriers to induce human infections (**Figure 2**). Two of them belonged to the alpha-CoV genera (HCoV-229E and HCoV-NL63) while the other five CoVs [HCoV-OC43, HCoV-HKU1, severe acute respiratory syndrome (SARS-CoV), Middle East respiratory syndrome (MERS), and SARS-CoV-2] are beta-CoV (**Figure 2**) (Cui et al., 2019; Mostafa et al., 2020). The natural reservoirs of these seven CoVs are bats and rodents where the virus is replicating asymptomatically before spilling over to intermediate mammals to acquire adaptive substitutions that facilitate zoonotic transmission to humans (**Figure 2**).

In late December 2019, SARS-CoV-2 emerged in Wuhan, China and induced clusters of pneumonia cases which promptly transmitted around the globe to cause the COVID-19 pandemic (Al-Karmalawy et al., 2021). Although the exact zoonotic transmission pathway of SARS-CoV-2 is still under investigation, most of the genetic and phylogenetic analysis indicated that bats might be the origin of SARS-CoV-2. In fact, SARS-CoV-2 and a bat CoV (RaTG13) share 96.2% nucleotide identity, however, the receptor binding domain (RBD), which is critical for virus-receptor binding, of these viruses are divergent (Zhou et al., 2020). These findings suggest that bats might not be the immediate origin of SARS-CoV-2, and there might be an intermediate host where the virus could replicate and adapt to easily infect humans.

Based on metagenomic analysis, several studies identified SARS-CoV-2-like viruses that shared 85-92% nucleotide identity with SARS-CoV-2 in small mammals known as pangolin (*Manis javanica*) (Lam et al., 2020). Albeit the low percentage of nucleotide identity between pangolin-SARS-CoV-2 and human-isolated SARS-CoV-2, their RBDs showed 97.4% homology. Thus, pangolin cannot be excluded as a potential intermediate host for SARS-CoV-2.

SARS-CoV-2 is composed of four structural proteins: spike (S), envelope (E), membrane (M), and nucleocapsid (N). These proteins share high sequence similarity to the sequence of the corresponding protein of SARS-CoV, and MERS-CoV. The virus entry is mediated by recognition and binding of the S protein to the cellular angiotensin-converting enzyme 2 (ACE2) receptor (Zhang et al., 2020). SARS-CoV-2 genome also encodes two polyproteins (pp1a and pp1ab) from the ORF1a and ORF1ab, respectively, that are further processed by the viral proteases papain-like protease (PLpro) and main protease (Mpro or CLpro) into 16 nonstructural proteins (Nsp1-16) that are essential determinants of innate immunity antagonism, replication efficiency and viral pathogenicity (Jahirul Islam et al., 2023). At the 3′ end of the SARS-CoV-2 genome, there are coding regions for several accessory open reading frame (ORF) proteins, including ORF3a, ORF3b, ORF6, ORF7a, ORF7b, ORF8b, ORF9b, and ORF10 (Zandi et al., 2022).

## 3 Reverse zoonosis of influenza A/H1N1pdm09 viruses and its molecular determinants

Since the emergence of influenza A/H1N1pdm09 virus in 2009 in North America, the first influenza pandemic in the twenty-first century in humans, influenza A/H1N1pdm09 virus has been circulating and established among humans as one of the seasonal influenza viruses (Smith et al., 2009). On the other hand, several transmissions of the same virus lineage from humans to other species have been determined (Abdelwhab and Mettenleiter, 2023). Such transmission from humans to other mammals are so-called reverse zoonoses. Since 2009, influenza A/H1N1pdm09 virus has been frequently isolated worldwide from pigs, the mixing vessel host for reassortment of influenza viruses, indicating the re-introduction to swine populations (Howden et al., 2009; Moreno et al., 2010; Pasma and Joseph, 2010; Pereda et al., 2010; Song et al., 2010; Sreta et al., 2010; Welsh et al., 2010; Holyoake et al., 2011; Kim et al., 2011). Intriguingly, the evolution pattern of the HA genes from A/H1N1pdm09 viruses circulating in humans and pigs are substantially different (Khalil et al., 2021), indicating the different impact of the ecology of both swine and human influenza A/H1N1pdm09 viruses. Also, this suggests the importance of continuous surveillance activities of SIVs in pigs to prevent the re-introduction of antigenically different variants from pigs to humans.

**Figure 3:**
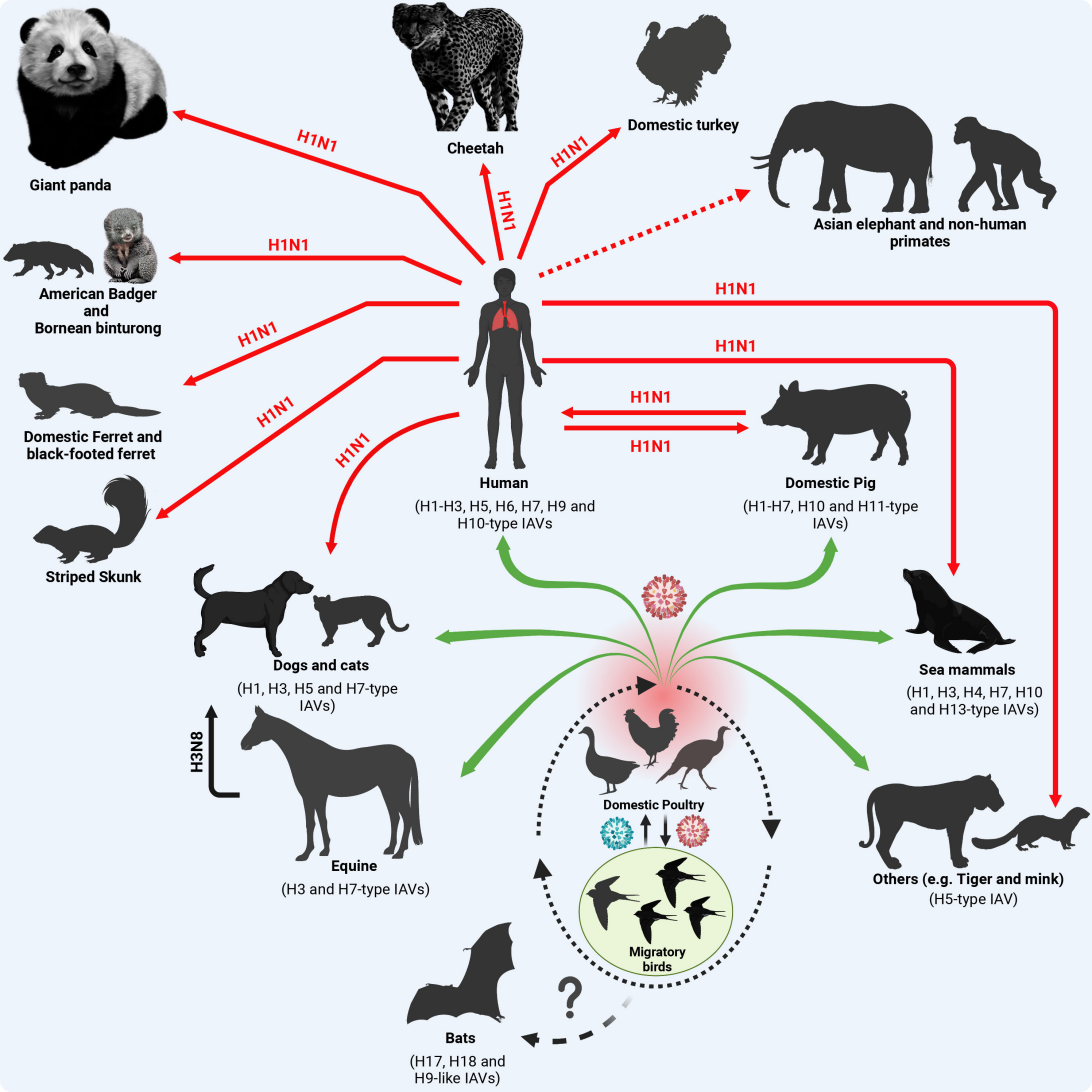
Origin of influenza A/H1N1pdm09 virus and reverse zoonosis in domestic and wildlife animals. Except for the BIVs, all IAVs are circulating in migratory birds as their natural reservoir that transmit the virus (green color virus) to the terrestrial and domestic birds at their stopover sites (dotted oval shape). Furthermore, the virus acquires essential adaptive mutations (red color virus) to cross species barriers and infect contact animals and humans (green arrows). In the case of influenza A/H1N1pdm09, the virus has been generated in swine following a multiple reassortment events between avian, human and swine influenza viruses (**Figure 1**) with distinct genetic constellation that enabled the virus to infect human causing a devastating pandemic and further transmit to contact domestic and wildlife animals. Red solid arrows refer to active virus detection. The dotted red arrow refers to serological evidence to virus exposure. This figure was created with BioRender.com.

In addition to swine, reverse zoonotic events of the influenza A/H1N1pdm09 virus were detected in other mammalian species (**Figure 3**). In brief, reverse zoonoses of influenza A/H1N1pdm09 virus were detected in captive giant panda in Hong Kong in 2019 (Martelli et al., 2019); and striped skunk in 2009/2010, 2013/2014, and 2015/2016 winter seasons (Britton et al., 2019) in Canada (Usui et al., 2021). Clinical and subclinical infections in cats and dogs with influenza A/H1N1pdm09 were also documented in different studies (Fiorentini et al., 2011; Su et al., 2014). Furthermore, serum antibodies against influenza A/H1N1pdm09 viruses were detected in pets (dogs and cats) in 2021 in Kyiv, Ukraine (Kovalenko et al., 2021). Beside its detection in domestic ferrets and giant anteaters (Nofs et al., 2009), the influenza A/H1N1pdm09 virus has been also detected in several wildlife species including Bornean binturong, American badger, and black-footed ferret (Schrenzel et al., 2011). The influenza A/H1N1pdm09 virus was also detected in mink in Europe, North America, and China (Åkerstedt et al., 2012; Clayton et al., 2022). In 2019, influenza A/H1N1pdm09 virus was detected via RT-PCR in captive cheetah showing respiratory signs of infection in a zoo in Japan (Usui et al., 2021). Serological and molecular detections of influenza A/H1N1pdm09 virus in domestic Asian elephants and non-human primates were reported in different countries (Karlsson et al., 2012; Paungpin et al., 2017). Interestingly, influenza A/H1N1pdm09 virus infections were also detected in domestic avian species (e.g. turkeys) in two breeder premises in the United Kingdom (UK) in late 2010 and early 2011 (Reid et al., 2012). This emphasizes the perspective that influenza A/H1N1pdm09 virus shifting towards mammalian hosts via improving its ability to bind or replicate in mammalian cells does not affect its ability to bind or replicate in avian cells.

Overall, these reverse zoonotic events imply the wide susceptible host range of infuenza A/H1N1pdm09 virus transmissions and diversity of virus evolution in different domestic and free-living wildlife host species. To enable influenza virus transmission from their ancestral natural reservoir or intermediate hosts to infect humans and further disseminate the human-to-human transmissible virus to other non-human animal species, the virus demands the acquisition of distinct and specific genetic markers. These adaptive changes improve the viral fitness in variable mammalian biological systems and their corresponding natural variations including body temperatures. Herein we highlight documented adaptive amino acid (aa) substitutions in the different viral proteins of influenza A/H1N1pdm09 virus responsible for adaptation to mammalian host(s).

### 3.1 Adaptive substitutions in polymerase basic 2 subunit

PB2 is one of the main components of the influenza vRNP complex made of PB2, PB1, PA, and NP that are essential for virus genome replication and gene transcription processes (Webster et al., 1992). The PB2 is the cap-binding subunit polymerase that enables the methylation and thus transcription of virus mRNA through acquiring the host messenger (m)RNA cap in a process called “Cap Snatching” (Guilligay et al., 2008). Additionally, PB2 has been known as a fundamental gene for influenza virus host adaptation. PB2 E627K is a main determinant host adaptive substitution that regulates virus polymerase activity, virus replication, and temperature sensitivity in a species-specific fashion. Glutamic acid (E) at position 627 in PB2 is an avian influenza virus signature that enables efficient virus polymerase activity, virus replication, and dynamics in avian species, whereas lysine (K) correlates with enhanced virus activities in mammalian species (Subbarao et al., 1993). Strikingly, the influenza A/H1N1pdm09 virus, even after continuous circulation in humans, still contains the avian signature E627 in PB2 which normally correlates with impaired virus replication in human cells (Fraser et al., 2009). Nevertheless, other adaptive substitutions have been acquired for compensating the absence of PB2 E627K aa substitution in influenza A/H1N1pdm09 virus (Mehle and Doudna, 2009). For instance, two aa substitutions in the PB2 of influenza A/H1N1pdm09 virus; serine (S) at position 590 and arginine (R) at position 591, called the SR polymorphism, were identified to be responsible for efficient polymerase activity and virus replication of influenza A/H1N1pdm09 virus in human cells (Mehle and Doudna, 2009). This SR polymorphism was identified in >20% of the sequences of SIV isolates in pigs but only after the emergence of a triple reassortant SIV in 1998-1999 (Webby et al., 2000; Olsen et al., 2006). Additionally, the SR polymorphism was determined to be only occurring when there is a E at position 627, which correlated with the PB2 E627 present in the influenza A/H1N1pdm09 virus (Mehle and Doudna, 2009).

### 3.2 Adaptive substitutions in polymerase basic 1 subunit

PB1 is the second component of the vRNP complex and is mainly responsible for polymerase extension during influenza virus replication (Perez and Donis, 2001). Several aa substitutions have been determined to enhance AIV adaptation in human and mammalian cells including, among others 336I, 361R, 486K, and 584Q into PB1; and 27I in PB1-F2 (Giria and Rebelo de Andrade, 2014). Also, aa substitutions 618D and 638D in PB1 have been described to promote PB1 activity after the genetic reassortment in the North American triple reassortant and influenza A/H1N1pdm09 viruses, respectively. Additionally, L298I, R386K, and I/A517V substitutions in PB1 have been described to putatively ameliorate the adaptation of influenza A/H1N1pdm09 virus in humans (Santos et al., 2023).

### 3.3 Adaptive substitutions in polymerase acidic subunit

PA is the third element of the influenza vRNP complex which has an imperative role in virus endonuclease activity that is essential in the Cap-snatching process and virus replication/transcription (Perez and Donis, 2001). Several aa substitutions, including A36T, T85I, G186S, L336M, E349G, and T552S, have been shown to enhance virus polymerase activity and replication in mammalian cells (Lutz et al., 2022). Also, aa substitutions T85I, G186S, and L336M have been described to increase virus adaptation in mammals through enhancing the PA binding to host RNA-binding protein (GRSF-1) that regulates viral mRNA cytosolic accumulation and translation efficiency (Lutz et al., 2022). Additionally, PA N321K substitution has been shown to enhance viral polymerase activity in human cells (Elderfield et al., 2014). In addition, the PA-X protein, produced from a ribosomal frameshift (+1) in the PA of IAV, contributes to improved viral replication and suppression of the host immune responses via enhancing virus-induced host shutoff activity (Gaucherand et al., 2019). Briefly, PA-X modulates the host immune response through the endonucleolytic domain that degrades the host mRNAs and thus suppresses the host gene expression (Clark et al., 2017; Nogales et al., 2017; Nogales et al., 2018b; Nogales et al., 2018a). Molecular studies showed that PA-X of early circulating influenza A/H1N1pdm09 viruses induced shut off to host gene expression, while this feature waned in the PA-X of recent circulating influenza A/H1N1pdm09 viruses (Nogales et al., 2018a). Genomic analysis of the PA-X from both early and recent influenza A/H1N1pdm09 viruses revealed four aa substitutions (V100I, N204S, R221Q, and L229S) in the PA-X of recent influenza A/H1N1pdm09 viruses that were responsible for affecting the shutoff activity induced by PA-X (Nogales et al., 2018a). Nevertheless, other compensatory substitutions in the NS1 of recent influenza A/H1N1pdm09 strains were described to allow the NS1 of influenza A/H1N1pdm09 virus to shutoff host gene expression, an function not present in viruses at the beginning of the pandemic, and therefore, compensate the lack of this function in the PA-X of recent influenza A/H1N1pdm09 viral isolates (see section 3.7) (Clark et al., 2017; Nogales et al., 2017; Nogales et al., 2018b; Nogales et al., 2018a).

### 3.4 Adaptive substitutions in hemagglutinin protein

HA glycoprotein is the main antigenic component of IAV that elicits the induction of host immune response and is responsible for binding to the host receptor and mediating virus entry to susceptible cells (Nerome et al., 1983). HA0 (neutral pH structure) is known as a typical class I fusion protein in which acid-induced refolding is irreversible (Parker et al., 2019), and is made of the HA1 subunit that contains the receptor binding domain (RBD), and the HA2 subunit that contains the fusion peptide. Following viral particle binding to host cell, the viral particle is internalized via endocytosis into the host cell cytoplasm. To initiate uncoating process and release the vRNP complexes into the cytosol, and then to the nucleus, the interior of the endosomes have a mildly acidic pH (pH 5–6) (Aganovic, 2023), causing protonation and resulting in a major conformational change in the viral HA, allowing the fusion of the HA2 subunit to fuse the membrane of the endosome with the membrane of the virus, resulting in the release of the viral genome into the cytoplasm of the infected cells (Di Lella et al., 2016). To this point, pH stabilization of HA is crucial for assessing viral host adaptation parameters including viral replication, pathogenesis, and transmissibility (Russell et al., 2018; Singanayagam et al., 2019; Aganovic, 2023; Tosheva et al., 2023). Moreover, HA stability has been recently investigated as a novel trait associated with the ability of IAVs to cross species barriers (Russell et al., 2018).

Species-specific aa substitutions are required to facilitate the entry of IAV to host cells and mediate low endosomal pH to allow membrane fusion (Vanderlinden and Naesens, 2014). The aa substitutions I32L, D97N, S185T, E374K, and S451N have been shown to enhance the affinity of influenza A/H1N1pdm09 HA glycoprotein to human α2-6 sialic acid receptors (Elderfield et al., 2014). Also, the E374K substitution enhances pH stabilization of influenza A/H1N1pdm09 virus HA in human cells (Yang et al., 2014). Overall, the evolution pattern of influenza A/H1N1pdm09 HA has been shown to render virus stability rather than antigenicity in human populations (Castelán-Vega et al., 2014).

### 3.5 Adaptive substitutions in the viral nucleoprotein

NP is one of the major structural proteins of IAVs and one of the main components of the vRNP complexes, in addition to its critical role in switching virus replication/transcription (Mostafa et al., 2018). Although NP is a relatively highly conserved protein among IAVs, several adaptive substitutions in NP have been shown to have a critical role in overcoming virus species barriers and rendering resistance to the host immune response (Mänz et al., 2013).

The myxovirus resistance protein 1 (Mx1/MxA), an interferon-induced GTPase that belongs to the dynamin superfamily of large GTPases, is one of the host-cell innate immune response mediators that has antiviral activity against several RNA viruses, including influenza (Haller and Kochs, 2011). During influenza virus infection, MxA forms tetramers and oligomers that assemble as barrier rings in the cytoplasm and hinder the translocation and function of vRNP complexes (Nigg and Pavlovic, 2015). The NP of influenza A/H1N1pdm09 virus has been shown to harbor aa substitutions, including E53D, R100V, P283L, Y289H, R305K, F313V, I316M, T350K, R351K, V353I, and Q357K; that confer virus resistance to MxA and, therefore, allow influenza A/H1N1pdm09 virus to evade host innate immune antiviral responses (Mänz et al., 2013).

### 3.6 Adaptive substitutions in neuraminidase protein

NA glycoprotein is the second major dominant antigenic component of IAVs influenza virus that is responsible for the release of progeny virions from infected cells through its NA activity. The aa substitutions V106I and N248D in the NA glycoprotein of influenza A/H1N1pdm09 virus have been shown to enhance viral stability through modifications in the pH tolerance (Elderfield et al., 2014).

### 3.7 Adaptive substitutions in viral non-structural protein 1

NS1 is the non-structural protein of IAVs, and it has two main functional domains: the N-terminal RNA binding domain, involved in binding to RNA; and the C-terminal effector domain that regulates multiple functions including antagonizing the host antiviral immune IFN responses through many pathways (Kochs et al., 2007; Nacken et al., 2014; Petersen et al., 2018). Binding to the cleavage and polyadenylation specificity factor 30 (CPSF30) is the one of the main pathways used by IAV NS1 to block host mRNA transcription, including IFN-induced genes encoding for different proteins with antiviral activity (Ramos et al., 2013). Notably, the NS1 of influenza A/H1N1pdm09 virus lacks the ability of binding to the CSPF30 (Hale et al., 2010). However, certain aa substitutions (R108K and G189D) have been shown to allow NS1 binding to CSPF30 and thus inhibit host mRNA nuclear export. Intriguingly, although the majority of influenza A/H1N1pdm09 viruses encode R and G residues at positions 108 and 189, respectively, 108K and 189D were also encoded to a lesser extent in the influenza A/H1N1pdm09 viruses (Huang et al., 2021). Notably, influenza A/H1N1pdm09 viruses found later during the pandemic were shown to contain aa substitutions, including E55K, L90I, I123V, E125D, K131E, and N205S; that allow NS1-mediated inhibition of host gene expression (Clark et al., 2017). These aa changes allow later influenza A/H1N1pdm09 viruses in the pandemic to induce cellular shutoff to compensate those affecting the ability of PA-X of later pandemic influenza A/H1N1pdm09 strains (see section 3.3) (Clark et al., 2017; Nogales et al., 2017; Nogales et al., 2018b; Nogales et al., 2018a). These findings suggest that inhibition of host gene expression by influenza A/H1N1pdm09 virus, and most likely other IAVs, is most likely subject to a balance between NS1 and PA-X which can determine virus pathogenesis and fitness. Notably, manipulating the ability of influenza NS1 and PA-X to induce cellular shutoff could be explored to generate attenuated forms of the virus for their potential use as live-attenuated vaccines (Nogales et al., 2017).

## 4 Reverse zoonosis events of pandemic SARS-CoV-2 and molecular determinants of its zooanthroponotic potential

**Figure 4:**
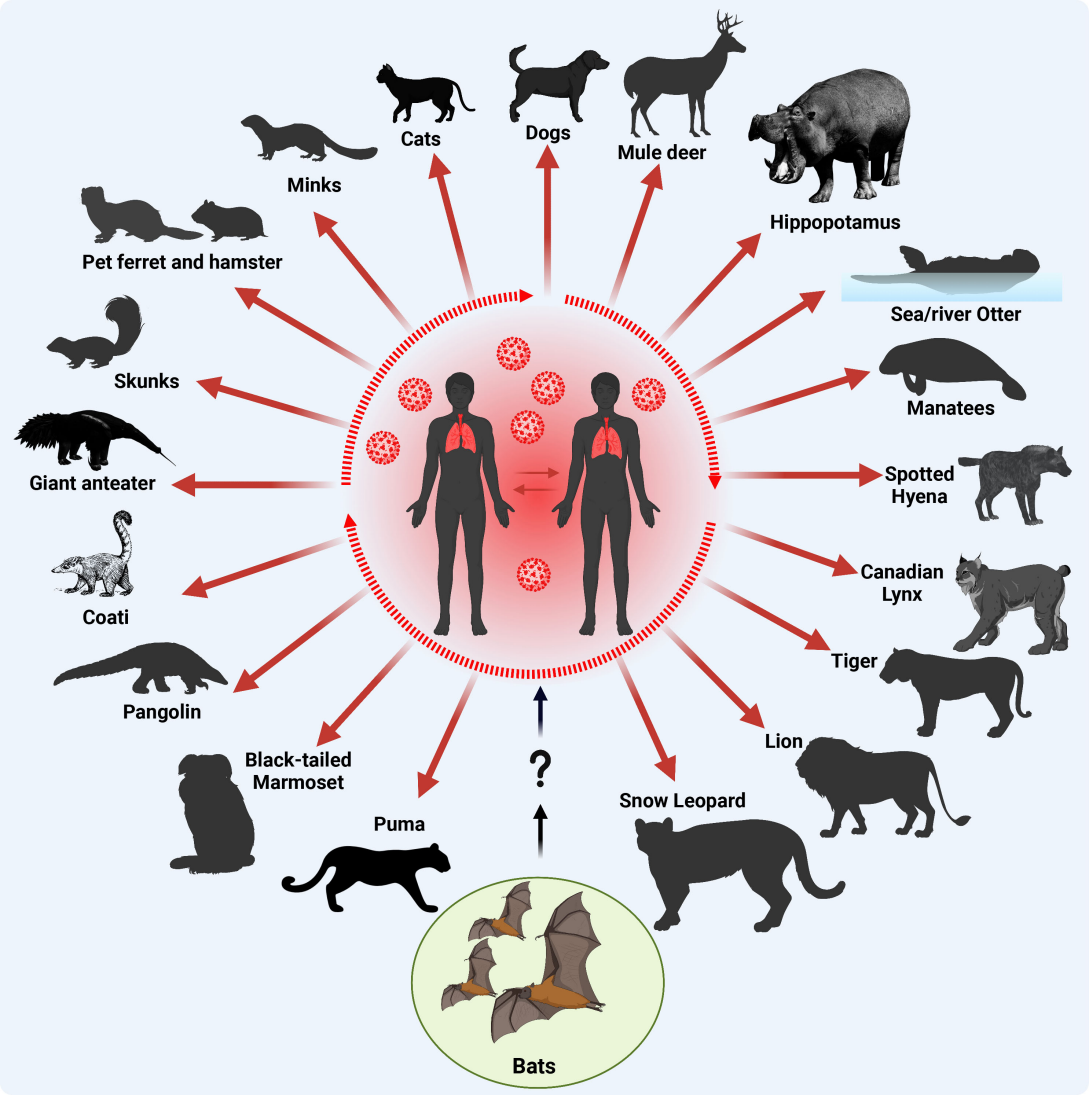
Origin of SARS-CoV-2 and documented reverse-zoonotic events. Following the transmission of SARS-CoV-2 into humans via uncertain intermediate host (?), most likely pangolins, the virus circulated in the human population acquiring adaptive substitutions to improve human-to-human transmission (a main criterion for a pandemic, dotted oval shape) and further transmitted to various contact domestic mammals, and free-living wildlife animals. Red arrows indicate SARS-CoV-2 reverse zoonotic/zooanthroponosis events. This figure was created with BioRender.com.

Since its emergence in late 2019, SARS-CoV-2 was subjected to multiple evolutionary events resulting in the emergence of several variants of concern (VOC) with remarkable positively selected aa substitutions in the surface S protein (**Table 1**). In March 2023, the devastating scale of VOC was narrowed by the European Centre for Disease Prevention and Control (ECDC) after de-escalating the rarely circulating variants (BA.2-BA.5) (**Table 1**) (Cocherie et al., 2022; ECDC, 2023). Currently, a few variants that are either variants of interest (VOI) or variants under monitoring (VUM) are circulating with comparable impact on transmissibility, immunity, and virulence to the ancestor omicron variants (**Table 1**). Meanwhile, SARS-CoV-2 could transmit from infected humans to a variety of pets and wildlife animal species, including cats, dogs, mink, lions, tigers, and others (**Figure 4**). This wide host range tropism of SARS-CoV-2 suggests that the virus is already well-adapted to infect different mammalian species and it can further acquire distinct species-specific substitutions following its human-to-animal transmission to fulfill new host adaptation requirements and improve viral fitness (Damas et al., 2020; Tan et al., 2022). SARS-CoV-2 binds primarily to the ACE2 receptor on the surface of the host cell via its S protein (Damas et al., 2020). Remarkably, the ACE2 receptor is highly conserved among different mammalian species (Damas et al., 2020; Lan et al., 2020). Consequently, the aa substitutions that enhance receptor binding affinity in human might reflect comparable effects in other mammalian species (**Table 1**).

**Table 1:** SARS-CoV-2 variants and their distinct aa substitutions in the S protein from 2020 until May 2023.

Strain/Variant		Country of origin	Year of detection	Maker S aa substitutions	Impact on transmission	Current Status	Reference
WHO	Lineage						
Alpha	B.1.1.7	UK	2020	N501Y; D614G; P681H; E484K	Increased	De-escalated variant	(Davies et al., 2021)
Beta	B.1.351	South Africa	2020	K417N; E484K; N501Y; P384L; E516Q; D614G; A701V	Increased		(Tegally et al., 2021)
Gamma	P.1	Brazil	2020	K417N; E484K; N501Y; D614G; H655Y	Increased		(Faria et al., 2021)
Epsilon	B.1.427	USA	2020	L452R; D614G	ND		(Deng et al., 2021)
Eta	B.1.525	Nigeria	2020	E484K; D614G; Q677H	ND		(Jangra et al., 2021; Zhao et al., 2021)
ND	C16	Unknown	2020	L452R; D614G	ND		(Jangra et al., 2021)
Iota	B.1.526	USA	2020	L452R; D614G, S477N; E484K; A701V	ND		(Jangra et al., 2021)
Delta	B.1.617.2	India and UK	2020	L452R; T478K; K417N; D614G; P681R; E484X; Q613H; Q677H	Increased		(Kemp et al., 2022)
Lambda	C.37	Peru	2020	L452Q; F490S; D614G	ND		(Romero Pedro et al., 2021)
ND	C.36	Egypt	2020	L452R; D614G; Q677H	ND		(Deng et al., 2021)
ND	A.23.1	UK	2020	V367F; E484K; Q613H	ND		(Jangra et al., 2021)
ND	A.27	Unknown	2020	L452R; N501Y, A653V; H655Y	Increased		(Davies et al., 2021)
ND	A.28	Unknown	2020	E484K; N501T; H655Y	ND		(Jangra et al., 2021)
ND	B.1.1.519	Mexico	2020	T478K; D614G	ND		(Deng et al., 2021)
Zeta	P.2	Brazil	2021	E484K; D614G	ND		(Jangra et al., 2021)
Theta	P.3	The Philippines	2021	E484K; N501Y; D614G; P681H	Increased		(Davies et al., 2021)
ND	B.1.616	France	2021	V483A; D614G; H655Y; G669S	ND		(Fillâtre et al., 2022)
Kappa	B.1.617.1	India	2020	L452R; E484K; D614G; P681R	Increased		(Pascarella et al., 2021)
ND	B.1.617.3	India	2021	L452R; E484Q; D614G; P681R	Increased		(Davies et al., 2021)
ND	B.1.620	Unknown	2021	S477N; E484K; D614G; P681R	ND		(Jangra et al., 2021)
Mu	B.1.621	Colombia	2021	R346K; E484K; N501Y; D614G; P681H	ND		(Jangra et al., 2021)
ND	B.1.1.7	UK	2021	L452R; S494P; N501Y; D614G; P681H	Increased		(Davies et al., 2021)
ND	B.1.1.318	Unknown	2021	E484K; D614G; P681H	ND		(Jangra et al., 2021)
ND	AT.1	Russia	2021	E484K; D614G; N679K; Ins679GIAL	ND		(Jangra et al., 2021)
ND	AV.1	UK	2021	N439K; E484K; D614G; P681H	ND	De-escalated variant	(Jangra et al., 2021)
ND	AY.4.2	UK	2021	L452R; T478K; D614G; P681R; A222V; Y145H	Increased		(Angeletti et al., 2022)
ND	C.1.2	South Africa	2021	D614G; E484K; H655Y; N501Y; N679K; Y449H	Increased		(Davies et al., 2021)
ND	B.1.640	Congo	2021	D614G; F490R; N394S; N501Y; P681H; R346S; Y449N; Δ137-145	ND		(Galmiche et al., 2023)
Omicron	BA.1	South Africa	2021	Pan-omicron substitutions (POS) including G143D; G339D; S373P; S375F; K417N; N440K; S477N; T478K; E484A; Q493R; Q498R; N501Y; Y505H; D614G; H655Y; N679K; P681H; N764K; D796Y; Q954K; N969K **(+)** BA.1 specific S substitutions including A67V; Δ69-70; T95I; Δ143-145; Δ211/L212I/ins214EPE; S371L; G446S; G496S; T547K; N856K; L981F	Increased		(Hui et al., 2022; Lyngse et al., 2022)
	BA.2	South Africa	2021	POS **(+)** BA.2 specific S substitutions including T19I; L24S; Δ25-27; V213G; S371F; T376A; D405N; L452X; R408S	Increased		(Lyngse et al., 2022)
	BA.3	South Africa	2022	NSM	ND		(Desingu et al., 2022)
	BA.4	South Africa	2022	BA.2 **(+)** Δ69-70; L452R; F486V; R493Q	Increased		(Mohapatra et al., 2022; Tallei et al., 2023)
	BA.5	South Africa	2022	BA.2 **(+)** Δ69-70; L452R; F486V; R493Q	Increased		(Mohapatra et al., 2022; Tallei et al., 2023)
	BA.2.75	India	2022	BA.2 **(+)** W152R; F157L; I210V; G257S; D339H, G446S; N460K; Q493	ND	VOI	(Cao et al., 2022)
	CH.1.1	ND	2022	BA.2.75 **(+)** R346T; K444T; L452R; F486S	ND	VUM	(Uraki et al., 2023)
	XBB.1.5	USA	2022	BA.2 **(+)** Q183E; N460K; S486P; F490S	Increased	VUM	(Yue et al., 2023; WHO, 2023c)
	XBB.1.16	ND	2022	BA.2 **(+)** E180V; T478R; F486P	Increased	VOI	(Harris, 2023; WHO, 2023c)
	EG.5	ND	2023	XBB.1.5 (**+**) F456L and Q52H	Increased	VOI	(Parums, 2023; WHO, 2023c)
	BA.2.86	ND	2023	XBB.1.5 (**+**) I332V, K356T, V445H, N450D, N481K, A484K, and Δ483	Increased	VOI	(Yang et al., 2023; WHO, 2023c)

ND, Not determined; WHO, World health organization; Ins, Insertion; POS, Pan-omicron substitutions; NSM, No unique spike substitutions; substitutions were documented in structural and nonstructural viral proteins; (+): plus or in addition to.

Unlike mammalian ACE2 that demonstrate high similarity to human, avian species ACE2 has remarkable number of variations in the functional sites to bind SARS-CoV-2 S protein (Zhai et al., 2020). This finding is consistent with the experimental data showing that poultry are not susceptible to SARS-CoV-2 infection (Frazzini et al., 2022). In the same line, limited or rare surveillance programs for SARS-CoV-2 infections in contact animals and particularly wildlife during pandemics made it hard to conclude about possible aa substitutions that are supposed to facilitate the household transmission of the virus into pets and other wildlife mammals. Nevertheless, the transmissibility of the virus into these contact species could be affected by different individual variations including animal family group, age, health status, frequency of contact, and viral load in infected contact person; rather than specific aa substitutions in the virus (Hobbs and Reid, 2021; Meisner et al., 2022).

Throughout the pandemic and the evolution of hundreds of SARS-CoV-2 variants, the S protein acquired several aa substitutions to potentially enhanced the binding affinity of the virus to the ACE2 receptor and consequently facilitated cross-species virus transmissibility (**Table 1**). For instance, the D614G substitution that emerged at early stages of the COVID-19 pandemic, could increase the transmissibility of the virus among humans, and it’s possible it could also have similar effects from humans to other mammals. Various VOC and VOI, including alpha (B.1.1.7), Beta (B.1.351), gamma (P.1), delta (B.1.617.2), and the most prevalent omicron variants (BA.1-BA.2.86), accumulated multiple substitutions in their S protein that have been associated with increased transmissibility among humans (**Table 1**). The documented aa substitutions and their overall impact on virus transmission that may in turn affect the reverse zoonotic transmission of these variants from human to other contact animals are summarized in **Table 1**.

Within the 3.5 years of the COVID-19 pandemic, SARS-CoV-2 infections have been documented in dogs, cats, deer, hippopotamus, sea and river otter, manatees, spotted hyena, Canadian lynx, tiger, lion, snow leopard, puma, black-tailed marmoset, pangolin, coati, giant anteater, skunks, ferret, hamster, and minks (**Figure 4**) (Bosco-Lauth et al., 2020; Chandler et al., 2021; Mathavarajah et al., 2021; Melo et al., 2022; Padilla-Blanco et al., 2022; Klestova, 2023; Michelitsch et al., 2023; Vercammen et al., 2023). In an experimental study, authors demonstrated that both dogs and cats can be infected with SARS-CoV-2, although dogs do not seem to spread the virus as efficiently as cats (Bosco-Lauth et al., 2020). This suggests that some pets like cats could potentially play a role in spreading SARS-CoV-2. A more recent study suggested that the interspecies transmission of SARS-CoV-2 between humans and their household pet animals occurs on a regular basis (Michelitsch et al., 2023), and that SARS-CoV-2 infections in dogs, cats and pet Syrian hamsters are usually asymptomatic without remarkable clinical signs, making it difficult for the contact humans to observe pets being infected (Yen et al., 2022; Michelitsch et al., 2023). Therefore, basic hygiene measurements must be implemented while dealing with domestic cats or dogs during the COVID-19 pandemic to avoid potential mutual SARS-CoV-2 infections. At the molecular level, a recent genome-wide association study revealed that no single nucleotide variants (SNVs) were significantly associated with cats and dogs, potentially due to small sample sizes (Naderi et al., 2023). Despite a broad host range of permissive animals to SARS-CoV-2 infection, only three animal species are known to effectively transmit the virus: Syrian hamsters, mink and white-tailed deer (Markov et al., 2023). Until now, no animal-specific aa adaptations have been identified in the viral genome of SARS-CoV-2 circulating in Syrian hamsters (Markov et al., 2023). Nevertheless, an aa substitution in SARS-CoV-2 S protein, L18F, arose during a hamster outbreak in a warehouse in Hong Kong (Yen et al., 2022), with an ability to reduce antibody neutralization of the SARS-CoV-2 gamma variant infecting humans (McCallum et al., 2021).

The first occurrence of SARS-CoV-2 in mink occurred in two separate farms in the Netherlands between April and May 2020 (Oreshkova et al., 2020). Since then, multiple COVID-19 outbreaks were reported among minks in Europe and North America (Lu et al., 2021; Oude Munnink et al., 2021; Porter et al., 2023). In Denmark, the largest mink fur producer in the world, several outbreaks in minks were identified, resulting in the emergence of different SARS-CoV-2 clusters/variants (Krammer, 2020; Larsen and Paludan, 2020; Bayarri-Olmos et al., 2021). One variant “Cluster 5” of these Denmark mink SARS-CoV-2 variants attracted more attention because it was reported in humans within the mink outbreak region (Larsen and Paludan, 2020). This variant was characterized by five distinct aa substitutions in S protein, including Y453F, 69-70 deletion (Δ69-70), I692V, M1229I, and S1147L (Larsen and Paludan, 2020; Clayton et al., 2022). The Y453F substitution located in the RBD domain of SARS-CoV-2 S protein was found to be fundamental for efficient binding of the viral S protein and the mink ACE2 receptor (Ren et al., 2021).

In the United States of America (USA), the Animal and Plant Health Inspection Service (APHIS) has documented SARS-CoV-2 outbreaks in 18 mink farms from August 2020 to November 2023, using PCR (16 farms) or immunological antibody (2 farms) tests (Eckstrand et al., 2021; APHIS, 2023). In Europe, a COVID-19 outbreak in Danish mink farms was documented in June 2020, suggesting that minks can transmit the virus to contact uninfected minks (Boklund et al., 2021). By characterizing the SARS-CoV-2 in mink and contact humans, data suggests that infected minks could transmit the virus readily to contact minks and farm personnel (Oude Munnink et al., 2021). The SARS-CoV-2 in this mink outbreak was found to carry five distinct substitutions/deletion in the S protein (ΔH69-V70, Y453F, D614G, I692V, M1229I) (Hoffmann et al., 2021). By comparing the genomic landscapes of SARS-CoV-2 isolated from animal species to that in humans, one study identified 5 animal-specific S and non-S adapted substitutions in minks: NSP9_G37E, S_F486L, S_N501T, S_Y453F, and ORF3a_L219V (Tan et al., 2022). Fortunately, the mink-adaptative substitutions in the S protein were unlikely to increase viral pathogenicity in humans, as Y453F attenuates the replication of the virus in human cells and could only lead to minimal antigenic impact or partial immune escape potential (Tan et al., 2022; Zhou et al., 2022). From January 2021 to July 2021, SARS-CoV-2 was identified in fourteen Polish mink farms. These mink farms were infected with four different SARS-CoV-2 variants (Domańska-Blicharz et al., 2021; Domańska-Blicharz et al., 2022). The etiologic agents of these outbreaks belong to eight different variants including 20B (two farms), alpha (one farm) delta (eight farms), and omicron (one farm) (Domańska-Blicharz et al., 2023). Between September 2022 and January 2023, another three mink farms were reported positive for SARS-CoV-2 (Domańska-Blicharz et al., 2023). The mink’s SARS-CoV-2 genome in this outbreak were characterized by aa substitutions in S proteins, including W64L, F486L, N501T, T572I, S929I, and Δ140–143 (Domańska-Blicharz et al., 2023). Interestingly, aa substitutions F486L and N501T have been previously reported as animal-specific changes associated with SARS-CoV-2 circulation in minks (Tan et al., 2022; Domańska-Blicharz et al., 2023). The high evolutionary rates of SARS-CoV-2 in minks in response to greater selective pressures in the new host are more than any other farmed animal species and could permit viral transmission among humans and other contact animals on mink farms (Peacock and Barclay, 2023; Porter et al., 2023). However, no clear evidence suggests that these adaptive aa substitutions may be a significant factor in SARS-CoV-2 zoonosis and transmission from minks to contact humans. A recent study revealed that the zooanthroponotic transmission of SARS-CoV-2 was associated with three SNVs (non-synonymous mutations) in minks, including ORF3a_L219V, Nsp9_G37E and S_N501T (Naderi et al., 2023).

In parallel, multiple outbreaks of SARS-CoV-2 among wild white-tailed deer (WTD) have been documented initially in the USA as a wildlife host for SARS-CoV-2 with 40% seroprevalence among sampled free-ranging WTD across four states (Chandler et al., 2021; Hale et al., 2022; Kuchipudi et al., 2022). Shortly after, active viral infections with different SARS-CoV-2 variants and high seroprevalence among free-ranging deer were detected in different localities in the USA (Hale et al., 2022; Roundy et al., 2022; Vandegrift et al., 2022; Caserta et al., 2023; McBride et al., 2023). Interestingly, the viral genome sequences from WTD are highly divergent from human-derived SARS-CoV-2 sequences with large nucleotide sequence variations across the genome, probably due to virus circulation and evolution within the deer population as a response to host adaptation (Caserta et al., 2023). Interestingly, several studies have revealed higher C-to-T bias in the SARS-CoV-2 genome from infected deer, which may reflect an evolutionary adaptation to APOBEC1 (Pickering et al., 2022; McBride et al., 2023; Naderi et al., 2023), a family of evolutionarily conserved cytidine deaminases that deaminates deoxycytidine in single-stranded DNA (ssDNA) and edits messenger RNAs (C-to-U editing) (Salter et al., 2016; Naderi et al., 2023).

The evolutionary rates of alpha and delta SARS-CoV-2 variants in WTD were shown to be faster and higher by 3 and 2.7 times than in humans, respectively (McBride et al., 2023). WTD infections with SARS-CoV-2 were associated with several aa substitutions in structural, non-structural and accessory ORF proteins including the variant specific recurrent substitutions in the S protein, such as the distinct L18F (delta), H69Y, N501Y (alpha, beta, gamma, omicron, mu) and T29I (alpha and delta) (McBride et al., 2023). Analysis of the whole genome sequences of alpha SARS-CoV-2 variants from infected WTD revealed that the zooanthroponotic transmission of SARS-CoV-2 in WTD was statistically associated with 26 SNVs (five intergenic mutations within the 5′ and 3′ UTRs, 12 synonymous mutations, and 9 non-synonymous mutations, including Nsp3_P822L, Nsp3_L1035F, Nsp3_S1437F, Nsp4_S386F, Nsp12_N507I, Nsp13_P77L, ORF5/M_I82T, ORF7a_T120I, and ORF10_L37F (Naderi et al., 2023). Consistently, other studies have identified the non-S aa substitution NSP3_L1035F as a more significantly deer-associated substitution (Tan et al., 2022), highlighting the importance of SARS-CoV-2 Nsp for virus fitness in the new host.

The phyloproteomic analysis of SARS-CoV-2 proteome sequences to investigate the variations in 16 non-human hosts (mink, cat, deer, dog, hyena, tiger, lion, gorilla, green monkey, Syrian hamster, leopard cat, fishing cat, bear cat, coati, ferret, and snow leopard) from 18 countries led to seven major divergent country-specific SARS-CoV-2 clades (Naderi et al., 2023). This study reported a number of high recurring (HR) aa substitutions in non-human hosts, including S_T19R, S_ΔH69-V70, S_G142D, S_E156G, S_ΔF157-R158, S_T478K, S_L452R, S_Y453F, S_F486L, S_N501T, S_D614G, S_P681R, S_D950N, N_D63G, N_S194L, N_R203K, N_G204R, N_G215C, N_D377Y, M_I82T, Nsp1_ΔM85, Nsp2_T85I, Nsp2_A192V, Nsp3_A488S, Nsp3_P1228L, Nsp3_L1244F, Nsp3_ΔN1263, Nsp3_P1469S, Nsp4_V167L, Nsp4_T492I, Nsp6_T77A, Nsp9_G37E, Nsp12_P323L, Nsp12_T739I, Nsp12_G671S, Nsp13_P77L, Nsp14_A394V, ORF3a_H182Y, ORF3a_Q57H, ORF3a_L219V, ORF3a_S26L, ORF7a_V82A, ORF7a_T120I, and ORF7b_T40I (Naderi et al., 2023). The contributing role of the substitutions in non-S proteins including the Orf1ab-derived Nsps, structural proteins, and accessory ORF genes in mediating virus zooanthroponotic and zoonotic potential is still unclear. Interestingly, this study could provide evidence that the occurrence of the non-human SARS-CoV-2 variants in humans is possible, emphasizing the zooanthroponotic and zoonotic transmission events between human and non-human hosts (Naderi et al., 2023).

## 5 Adverse impacts of reverse zoonosis on potential prophylactic, therapeutic interventions, and virus evolution

Zoonotic viruses transmit among hosts and can undergo strong and stringent adaptive selection to improve their fitness in their new niche (Mostafa et al., 2018; Mostafa et al., 2020; Al-Karmalawy et al., 2021; Markov et al., 2023). Although susceptible mammalian hosts have host cell receptor similarities in type, affinity, and abundance, the gradual improvement of viral fitness and transmission ability could be associated with a continuous evolution of antigenicity resulting in altered vaccine efficacy and resistance to limited antiviral treatment (Morris et al., 2020; Markov et al., 2023; Wong and Lal, 2023).

The seasonal human influenza vaccines are either propagated in specific-pathogen free (SPF) chicken embryonated eggs (avian-origin) or certified cell culture cell lines, including Madin-Darby canine kidney (MDCK) and African green monkey (Vero) cells with predominant α2,3-linked (avian-type) sialic acid receptor (Mostafa et al., 2016; Mostafa et al., 2018). The passaging of human influenza vaccine strains with an absolute affinity towards α2,6-linked (mammalian-type) sialic acid receptor in avian or avian-like mammalian systems is occasionally associated with low vaccine effectiveness due to adaptive aa substitutions in or around important antigenic sites of the immunogenic viral surface proteins HA and NA (Mostafa and Pleschka, 2018; Skowronski and De Serres, 2018; Liang et al., 2022).

In addition, the detection of AIV of H5-, H7-, and H9-subtypes in poultry carrying human adaptive aa substitutions in their PB2 segments, including G590S/Q591R and E627K, together with antiviral resistance markers that confer resistance to NA inhibitors, including H275Y and N295S, or M2 blockers (e.g. S31N) without apparent prior adaptation into mammals (Hossain et al., 2021), suggest possible reverse zoonotic transmission of these AIV strains from infected humans or mammals to poultry. This may explain the increasing abundance of antiviral resistance to adamantanes (M2) and neuraminidase (NA) inhibitors, and the high risk to human public health in possible outbreaks and/or potential pandemic situations (Lampejo, 2020; Jones et al., 2023).

Following influenza virus transmission from human to infect contact and wildlife animal species, moving through various biological systems, the new host animal will act as an additional reservoir for the virus that may yield an increased rate of adaptive aa substitutions or provide a new vessel (e.g. swine) to mix the genetic materials of two invading viruses (Chastagner et al., 2019; Rajao et al., 2019; Abdelwhab and Mettenleiter, 2023). This bidirectional transmission of influenza virus ends up with new virus variant(s) with unprecedented characteristics in humans.

Similarly, the transmission of SARS-CoV-2 between animal species could be associated with an increased rate of aa substitutions to adapt to the new hosts, resulting in adverse impacts on currently available vaccines and/or approved antiviral therapies (Hoffmann et al., 2021). In addition, CoVs have been shown to have high probability of recombination (Focosi and Maggi, 2022). Therefore, the wide host range of SARS-CoV-2 circulation among animal species with other CoVs might facilitate virus recombination with any of these CoVs following co-infection of the same host cell (Focosi and Maggi, 2022).

## 6 Conclusion

The wide spectrum of pandemic viruses, including influenza A/H1N1pdm09 virus and SARS-CoV-2, is alarming national and international health organizations to carefully follow up and control animal-to-human, human-to-human, as well as bidirectional human-to-animal zooanthroponosis transmission events. Pets and other animals that share household with infected humans, or farm animals, including minks, could be a persistent reservoir of these viral infections upon establishment of mild or non-asymptomatic infections, giving rise to potential new genetic reassortment, recombination, and evolution events, in addition to drug resistant and immune-escape variants. For these reasons, contact animals that are exposed to viral reverse zoonosis must be closely monitored in households, during transportation, and in wildlife since they could represent a new source of new zoonotic events to humans. Importantly, one of the major limitations in controlling viral pathogen zoonosis and zooanthroponosis includes the lack of a “One Health” concept, hindering an effective collaboration or coordination between animal and human health sectors in some areas with unusual habits with domestic pets and undomesticated animal species. Until now, we do not have solid background about the molecular determinant(s) of the zooanthroponosis of new emerging pandemic SARS-CoV-2 strains in most documented non-human hosts due to shortage in surveillance and the limited sample sizes. One Health surveillance strategy throughout different continents is more efficient and more sustainable than scattered efforts to monitor zoonosis and zooanthroponosis and control them at their first instance. Eventually, new and effective prophylactic and therapeutic countermeasures against newly emerging viral variants due to recurrent zoonosis and zooanthroponosis events must be developed and readily available. One limitation of this review is that most of the discussed data were mainly derived from European and North American countries where they have facilities and knowledge to follow up and characterize zoonosis and zooanthroponosis events.

## Author contributions

Conceptualization of the review, AK, LM-S and AM. Writing—original draft preparation, AK, LM-S and AM. Writing—review and editing, AK, LM-S and AM. All authors contributed to manuscript revision, read, and approved the submitted version.
